# Cyberbullying among Finnish adolescents – a population-based study

**DOI:** 10.1186/1471-2458-12-1027

**Published:** 2012-11-23

**Authors:** Pirjo L Lindfors, Riittakerttu Kaltiala-Heino, Arja H Rimpelä

**Affiliations:** 1 School of Health Sciences, University of Tampere, Tampere, Finland; 2 Medical School, University of Tampere, Tampere, Finland; 3 Department of Adolescent Psychiatry, Tampere University Hospital, Tampere, Finland

**Keywords:** Adolescents, Cyberbullying, Witnessing cyberbullying

## Abstract

**Background:**

Cyberbullying, threatening or harassing another via the internet or mobile phones, does not cause physically harm and thus the consequences are less visible. Little research has been performed on the occurrence of cyberbullying among adolescents or the perception of its seriousness. Only a few population-based studies have been published, none of which included research on the witnessing of cyberbullying. Here, we examined exposure to cyberbullying during the last year, and its frequency and perceived seriousness among 12 to 18-year-old adolescents in Finland. We studied four dimensions of cyberbullying: being a victim, bully, or both victim and bully of cyberbullying, and witnessing the cyberbullying of friends.

**Methods:**

Self-administered questionnaires, including four questions on cyberbullying, were mailed to a representative sample of 12-, 14-, 16-, and 18-year-old Finns in 2009 (the Adolescent Health and Lifestyle Survey). The respondents could answer via the internet or paper questionnaire.

**Results:**

The number of respondents was 5516 and the response rate was 56%. Girls more often than boys reported experiencing at least one dimension of cyberbullying during the last year. The proportion was highest among 14-year-olds and lowest among 18-year-olds of both sexes. Among girls, the most commonly encountered dimension was witnessing the cyberbullying of friends (16%); and being a victim was slightly more common than being a bully (11% vs. 9%). Among boys, an equal proportion, approximately 10%, had been a victim, a bully, or had witnessed cyberbullying. The proportion of bully-victims was 4%. Serious and disruptive cyberbullying was experienced by 2% of respondents and weekly cyberbullying by 1%; only 0.5% of respondents had been bullied weekly and considered bullying serious and disruptive.

**Conclusions:**

Adolescents are commonly exposed to cyberbullying, but it is rarely frequent or considered serious or disruptive. Cyberbullying exposure differed between sexes, such that girls more often than boys witness cyberbullying of friends and boys more often act as the bully than girls. In future studies, the witnessing of cyberbullying and its consequences should be taken into account.

## Background

Cyberbullying, threatening or harassing another via the internet or mobile phones, occurs in several forms [1-3]. Compared to traditional bullying, cyberbullying has several specific features that may intensify its harmful effects, including the difficulty in escaping from the bullying, the magnitude of the potential audience, the anonymity of the bully, and the ability to attack at any time and any place. On the other hand, cyberbullying does not cause physical harm, making its consequences less visible, and nasty text messages or e-mails can be easily and quickly deleted [4-7]. Research on cyberbullying, its consequences, and seriousness, is scarce and only a few population-based studies have been published [3,6,8-11].

Among Western adolescents, the prevalence of cyberbullying victims (cybervictim) varies from 9% to 34% and that of cyberbullying bullies (cyberbullies) varies from 4% to 21% [1,4,5,7,9]. In a recent review [2], Tokunaga concluded that 20% to 40% of adolescents experience cyberbullying at least once in their lifetime and evidence indicates that the number of cybervictims is growing [5,12]. The definitions, measures, and methodology vary widely across studies and contribute to the inconsistencies of the findings [13].

New communication technologies may expose new groups of adolescents to bullying who might not be exposed to traditional face-to-face bullying, and provide a new means to bully those who have also been bullied by traditional methods [1]. Many cybervictims are also traditional victims, and, correspondingly, many cyberbullies are traditional bullies. In addition, bullying can spread across platforms such that an adolescent may be bullied by several routes simultaneously [5,14]. The two groups, namely cyberbullies and cybervictims, can overlap [4,6].

Research findings of the relationship between age and victimization by cyberbullying are inconsistent. Some studies show a decrease in cyberbullying with older age [4,5,8,15], while a majority of studies show no association with age [2]. Research findings regarding sex differences in the patterns of bullying are also mixed. Some studies show that girls are more likely to be cyberbullied [4,5,8], while others report no sex differences [9,14,16]. Some studies report that boys are cyberbullies more often than girls [3,12].

Cyberbullying and victimization are important adolescent health issues. The studies of cyber victimization report an association with concurrent psychosocial difficulties and risk factors such as distress, depressive mood, substance use, school conduct problems, or low caregiver-adolescent connectedness [5,6,10,14,15,17-19]. It is not clear, however, whether these symptoms are antecedents or consequences of cyberbullying, because the causality may be bidirectional [20]. The bully-victim group is considered the most problematic in terms of mental health and psychosocial problems [6].

Measures and methodology used to examine the seriousness of bullying experiences varies. Ybarra et al. found that 38% of harassed youth reported distress as a result of a bullying incident [18]. Wolak et al. studied different qualities of bullying experiences and found that 30% of the adolescents reported bullying as being very or extremely upsetting, 24% as very or extremely frightening, and 22% as very or extremely embarrassing [12]. In a pan-European study [8], most children reported being upset by online bullying and one-third report being very upset.

Cyberbullying occurs either as a group function or within group online communication environments [21]. Little attention, however, has been paid to the bystanders of cyberbullying. Bystanders can be either an active part of the problem by encouraging and supporting the bully or a passive part by watching and witnessing bullying, and doing nothing to stop it. On the other hand, some researchers believe that cyberbystanders, especially friends, play an important role in preventing acts of bullying by providing support to the victim [21,22].

Some studies have examined a broader context and different forms of bullying and victimization [11,23], but to our knowledge few studies have examined exposure in terms of witnessing cyberbullying and none of them evaluated population-based data. Patchin and Hinduja reported that 47% of 384 respondents answered “yes” to the following question: “Have you ever seen other kids bullied online”[16]. Li found that more than half of student respondents admitted knowing someone who had been cyberbullied [24]. Wolak et al. reported that approximately 30% of respondents were with friends when cyberbullied [12]. Evidence suggests that cybervictims are likely not to tell anybody about their cyberbullying experience or if they know of someone having been bullied. If they choose to tell, friends are told most often and parents second most often [9,10,16,17,25].

Exposure to cyberbullying by witnessing a friend being cyberbullied deserves attention, as witnessing cyberbullying may also be traumatizing. Bystanders who have witnessed face-to-face bullying without direct involvement present with increased incidence of psychiatric symptoms, and the incidence increases if the victim is a friend of the bystander [26]. This may hold true for witnessing cyberbullying as well, and thus exposure by witnessing warrants more attention.

The aim of the present study was to examine exposure to cyberbullying among 12 to 18-year-old adolescents. Here, cyberbullying refers to bullying via the internet or mobile phone, and exposure to one of four dimensions of the phenomenon: being a victim of cyberbullying, being a cyberbully, being both a cyberbully and a cybervictim, and having witnessed cyberbullying of friends. We first studied the proportion of adolescents that experienced cyberbullying during the preceding year and how serious and disturbing it was perceived to be. Second, we studied the proportion of respondents that had been a bully or a bully-victim. Finally, we studied whether adolescents had witnessed cyberbullying of their friends. Witnessing cyberbullying here means that one gets to know or observes that a friend or friends are being bullied.

## Methods

This study is based on the nationwide Adolescent Health and Lifestyle Survey, conducted biennially in Finland since 1977. We used the data from 2009. Nationally representative samples of 12-, 14-, 16-, and 18-year-olds were obtained from The Population Register Centre by selecting all Finns born on certain days in June, July, and August. Mean ages of the respondents were 12.6, 14.6, 16.6 and 18.6 years. The Ethics Committee of the Pirkanmaa Hospital District approved the study protocol. Self-administered questionnaires were mailed in February, followed by three reminders to non-respondents. The respondents could answer either via the internet or mailed questionnaire. The number of respondents was 5516 and response rate was 56%. Of the respondents, 66% were girls. Boys in all age groups responded less often than girls and younger age groups responded more often than the older age groups. The response rates of the 14-year-old boys and girls were 50% and 68%, respectively, and of 18-year-old boys and girls, 36% and 61%, respectively. In all, 49% of the respondents used the e-form questionnaire. We used the Pearson chi-squared test to study the age and sex differences at the level of p less than 0.05.

The 12-page questionnaire included approximately 100 questions on socio-demographic background, health behavior, and health. In terms of cyberbullying, respondents were asked four questions, formulated as follows: 1) During the last year, have you been bullied by mobile phone or via the internet? The four response options were: a) several times a week, b) approximately once a week, c) less than once a week, and d) not at all. Frequency measures were formulated on the basis of the Finnish School Health Promotion Study questions on traditional bullying. 2) If you have been bullied by mobile phone or via the internet, was the bullying serious and disturbed your life? The four response options were: a) bullying was very serious and disturbing, b) bullying was a little bit serious and disturbing, c) it was not serious and disturbing, and d) I have not been bullied. In the analyses, the responses to the seriousness of bullying questions were dichotomized as “very serious and disturbing or little bit serious and disturbing (serious and disturbing) vs. “not serious and disturbing”; 3) Have your friends or mates been bullied by mobile phone or via the internet during the last year? The five response options were: a) no, b) one of my friends has been bullied, c) 2 to 3 of my friends have been bullied, d) 4 or more of my friends have been bullied, and e) I do not have friends. Those who chose options b, c, or d in response to question 3 were defined as having witnessed cyberbullying of friends. 4) Have you bullied others or participated in bullying others by mobile phone or the internet during the last year? The response options were: a) several times a week, b) approximately once a week, c) less frequently than once a week, and d) I have not bullied.

## Results

The proportion of adolescents indicating exposure to at least one of the measured dimensions of cyberbullying (cybervictim, cyberbully, witnessing the cyberbullying of friends) was 23%. The proportion was higher among girls than boys. The proportion was lowest among 18-year-olds and highest among 14-year-olds of both sexes. Differences between age-sex groups were statistically significant (Table 1).

**Table 1:** Proportion of adolescents (%) who have been victims, bullies, bully-victims or witnessed cyberbullying of their friends during the last year by age and sex (N)

			**Boys**					**Girls**			**All**	**p sex**	**p age, sex**
	**12 yrs.**	**14 yrs.**	**16 yrs.**	**18 yrs.**	**Total**	**12 yrs.**	**14 yrs.**	**16 yrs.**	**18 yrs.**	**Total**			
	**(326)**	**(735)**	**(690)**	**(537)**	**(2288)**	**(356)**	**(999)**	**(962)**	**(911)**	**(3228)**			
Cyber-bullying during the last year													
Victim	10	13	9	9	10	14	14	11	7	11	11	.496	.000
Victim, bullying serious and disturbing	2	2	1	1	1	4	3	3	3	3	2	.000	.016
Bully	8	13	12	10	11	5	13	8	4	8	9	.000	.000
Bully-victim	3	6	4	3	4	2	5	3	2	3	4	.052	.000
Weekly cyberbullying													
Victim	1	1	1	1	1	1	1	1	1	1	1	.259	.819
Bully	0	1	1	1	1	0	0	1	0	0	1	.001	.003
Witnessed cyberbullying of friends during the last year	11	10	10	10	10	16	19	17	10	16	13	.000	.000

Among the girls, the most commonly encountered dimension of cyberbullying was witnessing the cyberbullying of friends (16%), and being a victim was slightly more common than being a bully (11% vs. 8%). Among the boys, an equal proportion, approximately 10%, had been a victim, a bully, or had witnessed cyberbullying of friends. Boys were bullies more often than girls (p<0.05). In both sexes and in all age groups, it was least common to be a bully-victim. Being a bully was least common among the 12-year-olds of both sexes and 18-year-old girls. Adolescents at the age of 14 years were most often exposed to cyberbullying (Table 1). Among all respondents, 9% reported that one friend had been cyberbullied during the last year, 4% reported two to three friends, and 1% reported four friends or more.

Although it was fairly common to be victim or a bully, it was rare to be a victim or a bully weekly. Of the 9% that reported that they had cyberbullied others during the last year, only 0.5% had bullied others once a week or more often. Correspondingly, only 1% reported having been a victim weekly, although 11% had been bullied. Age and sex differences of being a victim weekly and being a bully weekly generally followed the same pattern as being a traditional bullying victim or a bully (Table 1). The proportion of adolescents who perceived cyberbullying as very serious and disturbing was very small (1%). The percentage remained small even when those who reported slightly serious and disturbing bullying were included. Girls reported serious and disturbing bullying more often than boys (Table 1). Finally, the overall proportion of adolescents who had been bullied weekly and considered bullying serious was only 0.5%.

## Discussion

The present findings indicate that the prevalence of cyberbullying is 11%. The prevalence of cyberbullies was 9% and that of cyberbullying victims 4%. These results are similar to those of previous population-based studies. [3,6,8,11,12]. The prevalence of witnessing cyberbullying was highest of the four dimensions: 13% of adolescents reported that their friends had been cyberbullied. To our knowledge, this study is among the first to report the prevalence of cyberbully witnessing in a large-scale population-based sample. Our findings suggest that an adolescent may be exposed to cyberbullying through various routes simultaneously, and exposure by witnessing friend’s cyberbullying should also be taken into consideration in further studies.

Although the prevalence of bullying was fairly high, the intensity of the cyberbullying experiences was low. Only 2% of respondents reported being either a victim or a bully at least once a week. This percentage was in line with previous studies reporting that 4,3% of adolescents are frequently (two or three times a month) involved in cyberbullying, either as victims, bullies [11]. The seriousness of the cyberbullying experiences was low. Among those who were cyberbullied, approximately 20% considered the bullying to be serious and disturbing. This percentage is somewhat lower than that in previous studies, which reported that around 30% of adolescents who had been bullied had experienced as being very or extremely upset [8,12,18]. The inconsistency of these results might be due to variations in the methodology or might reflect consequences of different types of cyberbullying, e.g., text message vs. video clip [17].

Our results also revealed a constant pattern in terms of age and sex differences concerning being a victim and bully. Both 14-year old boys and girls bullied others more frequently and were victims themselves. These results are consistent with those of the previous studies in which younger children were more often involved in bullying than older children [3-5,8,9]. Correspondingly, boys were bullies significantly more often than girls [3,13]. The age pattern fits well with the psychological and physiological changes related to the turbulence of puberty. The younger age group, comprising 12-year-olds, might not have discovered this form of bullying, and cyberbullying tends to decrease with age. Some studies, however, demonstrated no association between age and victimization by cyberbullying [2]. In terms of witnessing cyberbullying, we found a strong sex difference: 12 to 16-year-old girls were exposed to cyberbullying via friends almost twice as much as boys. Girls’ friendships are very much based on mutual sharing, support, and confidence, thus girl friends share problems, including bullying experiences, and can also obtain social support from each other for difficult experiences.

The present study has some limitations. The 1-year time period defined in the questionnaire should be cautiously evaluated in terms of the reliability of the results due to memory bias. In the questionnaire, we did not define cyberbullying. We did, however, explicitly mention the term “bullying”, although it was left to the individual respondent to define whether or not an action was cyberbullying. A recent study [13] suggested that this line of questioning is a reliable method. Involvement in bullying was measured by self-reports, and it has been suggested that a self-report survey method is likely to result in underreporting of victimization [27], as well as of being a bully, which is a socially undesirable behavior. Because we wanted to study the phenomenon at the population level, however, the use of a survey was the optimal method. We were also particularly interested in the adolescents’ own perception of the issue, because it is their perception that will influence their behavior and emotional reaction. The sample was representative of the entire country, but the low response rate may have caused a selection bias of the respondents. Adolescents with non-normative behavior, such as those who smoke vs nonsmokers, participate in surveys less often. We cannot exclude the possibility of a selection bias here, but the direction is not known.

A novel finding of the present study was that exposure to cyberbullying by witnessing is a noteworthy dimension that should be taken into consideration in creating a picture of the complex phenomenon of cyberbullying. To our knowledge, this is the first population-based study to examine cyberbullying from this perspective. Our data revealed that witnessing cyberbullying was the most prevalent dimension of cyberbullying and indicated that an adolescent may be exposed to cyberbullying through several routes simultaneously.

## Conclusions

The results of the present study suggest that although adolescents are commonly exposed to cyberbullying, it is only rarely frequent or considered serious and disturbing. Boys are more often bullies than girls. Witnessing cyberbullying of friends was the most common dimension of exposure to cyberbullying. Girls witness cyberbullying of friends more often than boys do. More research is needed to obtain a clear picture regarding the nature, extent, and risk factors of cyberbullying. Further, the dimension of witnessing cyberbullying of friends and its consequences should also be taken into account in studies of exposure to cyberbullying.

## Competing interests

The authors declare that they have no competing interests.

## Authors’ contributions

PL carried out the analysis and drafted the manuscript. RK-H participated in the design of the study and drafted the manuscript. AR conceived of the study and finalized the manuscript. All authors read and approved the final manuscript.

## Pre-publication history

The pre-publication history for this paper can be accessed here:

http://www.biomedcentral.com/1471-2458/12/1027/prepub
